# Do anesthetics and sampling strategies affect transcription analysis of fish tissues?

**DOI:** 10.1186/1471-2199-8-48

**Published:** 2007-06-08

**Authors:** Pål A Olsvik, Kai K Lie, Ernst M Hevrøy

**Affiliations:** 1 National Institute of Nutrition and Seafood Research, Nordnesboder 2, N-5005 Bergen, Norway

## Abstract

**Background:**

The aim of the current examination was to evaluate if sedation and anesthetic treatment techniques affect the quality of RNA extracted from liver, gill, head kidney and brain tissues in Atlantic salmon *Salmo salar *L. Blood parameters were measured and tissue specimens sampled in six groups of fish; one control group (0 minutes), two groups kept in pure seawater in 90 liter tanks for 30 and 120 minutes, two groups treated with the anesthetic isoeugenol for 30 and 120 minutes, and one group kept in pure seawater for 105 minutes and then anaesthetized with metacaine for 15 minutes. RNA quality was assessed with the NanoDrop ND-1000 spectrophotometer (260/280 and 260/230 nm ratios) and with the Agilent Bioanalyzer (28S/18S ratio and RIN data) in samples either preserved in liquefied nitrogen (N_2_) or in RNA*later*. In addition, the transcriptional levels of two fast-responding genes were quantified in gill and brain tissues.

**Results:**

The results show that physiological stress during sampling does not affect the quality of RNA extracted from fish specimens. However, prolonged sedation (2 hours) resulted in a metabolic alkalosis that again affected the transcriptional levels of genes involved in ionoregulation and respiration. In gills, *Na*^+^-*K*^+^-*ATPase α1b *was significantly downregulated and *hypoxia inducible factor 1 *(*HIF1*) significantly upregulated after two hours of treatment with isoeugenol, suggesting that this commonly used sedative affects osmo-regulation and respiration in the fish. The results also suggest that for tissue preservation in general it is better to flash-freeze fish specimens in liquefied N_2 _than to use RNA*later*.

**Conclusion:**

Prolonged sedation may affect the transcription of fast-responding genes in tissues of fish. Two hours of sedation with isoeugenol resulted in downregulation of the *Na*^+^-*K*^+^-*ATPase α1b *gene and upregulation of the *HIF1 *gene in gills of Atlantic salmon. The quality of RNA extracted from tissue specimens, however, was not affected by sedation treatment. Flash-freezing of tissue specimens seems to be the preferred preservation technique, when sampling fish tissue specimens for RNA extraction.

## Background

To extract high quality RNA from tissues or cells is of crucial importance for downstream applications in molecular biology. Purity and integrity of the RNA are critical factors for most RNA-based assays, including transcription analysis. Traditionally, RNA quality was assessed by cuvette-based UV spectroscopy and ribosomal band electrophoresis, i.e. 28S/18S area ratios. Using spectrophotometer, a 260/280 nm ratio greater than 1.8 is usually considered to indicate acceptable RNA purity. The integrity of the RNA has normally been evaluated using formaldehyde agarose gel electrophoresis, a 28S/18S ratio of 1.8 – 2.0 is considered to be typical of high quality intact RNA. Today, many labs use the NanoDrop^® ^ND-1000 Spectrophotometer (NanoDrop Technologies) to accurately and reproducibly measure RNA in samples with volumes down to 1 μl and over a broad concentration range without dilution, and use microfluidic capillary electrophoresis with the Agilent 2100 Bioanalyzer (Agilent Technologies) to evaluate the RNA integrity. Provided with the Agilent 2100 expert software, the RNA integrity number (RIN) is a tool designed to automatically assign an integrity number to a eukaryote total RNA sample. With this tool, sample integrity is no longer determined by the ratio of the 28S/18S ribosomal bands, but rather by the entire electropherogram of the RNA sample, including the presence of degradation products [1,2]. The RIN is independent of sample concentration, instrument and analyst and therefore becoming a de facto standard for RNA integrity. Using these new instruments and techniques, old wisdom has been challenged. For example, Ambion now states that total RNAs with 28S/18S ratios of 1.0 or greater usually provide high quality intact RNA that perform well in a variety of applications [3].

Sedation and anesthesia of fish are often used in order to reduce stress levels in the animals during experimental sampling. Still, very little is known about the impact of choice of sedation and anesthetics on RNA quality and integrity from tissues sampled for transcription analysis. And further how RNA quality and integrity are affected by how the fish is handled during sampling, i.e. howling and crowding. Preanalytical steps like collection, storage and processing of fish samples may affects transcript stability, raising the possibility that partial degradation during cell lysis could cause a variable extent of bias in quantification of different transcripts [4]. Many sedatives and anesthetics have traditionally been used on fish, i.e. drugs, gases, hypothermia and electric current. Metacaine (C_9_H_11_NO_2_·CH_4_O_3_S, ethyl m-aminobenzoate methane sulfonate) is one of the most used local anesthetic in poikilotherm organisms. It is lipid soluble and either taken up through the gills by diffusion or by active transport. It is easily taken up and has a fast response on striated muscle, and acts by blocking Na^+^-channels [5]. The fish is immobilized very fast, allowing handling and metacaine has no persistent effects on fish physiology and behavior [6]. Recently, eugenol (CH_2_CH_2_CH_2_C_6_H_3_(OCH_3_)OH, 2-Methoxy-4-(2-propenyl)phenol) has been proposed used as an anesthetic on aquatic organisms. The active substance in eugenol is clove oil, derived from the stem, leaves or buds of the *Eugenia caryophyllata *tree. Eugenol as an anesthetic compound for fish, commercially sold as Aqui-S, contains 50% isoeugenol and 50% polysorbate 80. Iversen et al. [7] have shown that Aqui-S can be used as a sedative to keep salmonid fish calm during handling, loading and transfer to sea, as well as blocking plasma cortisol surge levels at dosages above 20 mg/L.

The objective of this study was to examine if speed of fish handling and choice of sedation and anaesthetization methods affect the quality of RNA extracted from Atlantic salmon *Salmo salar *tissue. Three groups of salmon, a total of 36 fish, were subjected to two different sedation/anaesthetization treatments or kept un-anaesthetized, and four tissues (liver, gills, head kidney and brain) sampled from each individual for RNA extraction after keeping the fish in 90 L tanks to induce crowding stress for 0, 30 and 120 min. The fish were killed by a blow to the head after either being kept in pure seawater, sedated by isoeugenol (Aqui-S) or anaesthetized with metacaine for 15 min. without isoeugenol pretreatment.

## Results

### Fish

**Figure 1:**
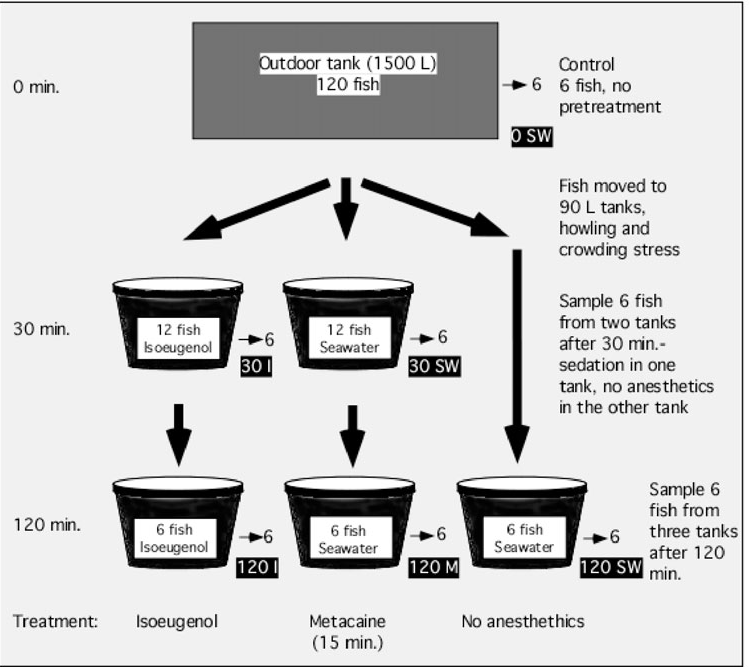
Experimental design. Six fish from six groups were sampled for analysis; 0 min. seawater control (0 SW), 30 min. seawater (30 SW), 30 min. isoeugenol (30 I), 120 min. seawater (120 SW), 120 min. metacaine (120 M) and 120 min. isoeugenol (120 I).

No fish died during the two-hour experimental period, but one fish was lost from each of the 120 min. metacaine and the 120 min. isoeugenol groups, lowering the number of fish from these groups to five for all the measured parameters. These two fish jumped out of the tanks during the treatment period. No significant differences in weight, length or condition factors were observed between the 6 groups (see Figure 1 for the experimental setup). On average the fish were weighing 455 ± 80 g, with an average length of 36.1 ± 1.8 cm. The average condition factor (K = weight (g)/length (cm)^3 ^× 100) was 0.96 ± 0.06. Only juvenile, no-mature fish were used in the experiment. None of the measurements were therefore differentiated based on gender.

### Blood/plasma

**Figure 2:**
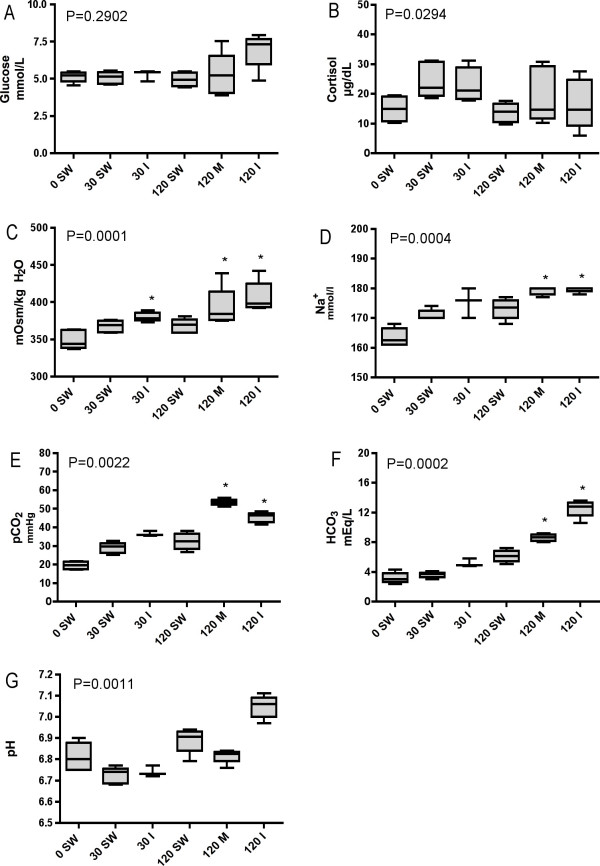
Blood and plasma parameters analyzed in the six different fish groups. Group identity: 0 min. seawater control (0 SW), 30 min. seawater (30 SW), 30 min. isoeugenol (30 I), 120 min. seawater (120 SW), 120 min. metacaine (120 M) and 120 min. isoeugenol (120 I). A) glucose, B) cortisol, C) osmolality, D) Na^+^, E) pCO_2_, F) HCO_3 _and G) pH. For cortisol (C) and osmolality measurements (D), n = 6 in all groups except 120 min. metacaine and 120 min. isoeugenol groups (n = 5). For the I-Stat measurements (A, D, E, F, and G), n = 6 in the 0 min. control group, n = 5 in the 30 min. group, n = 3 in the 30 min. isoeugenol group, n = 4 in the 120 min. and the 120 min. metacaine group and n = 5 in the 120 min. isoeugenol group. The horizontal line in the middle of the box plot show the median (50% percentile) of the sample. The top and bottom of the box show the 75^th ^and 25^th ^percentiles, respectively, whereas the top and bottom of the whiskers show the maximum and minimum values. An * denotes significant differences (P < 0.05) between the 0 min. control group and the other groups (Kruskal Wallis ANOVA).

Whole blood parameters analyzed with the I-STAT, cortisol levels and osmolality are shown in Figure 2. Kruskal-Wallis ANOVA P-values are shown in the figures. Due to problems with some of the I-STAT cartridges, the number of individual blood parameter measurements was lower than six in some of the groups (see Figure 2 caption text). In addition, two of the Na^+ ^measurements in the 120 min. metacaine group and one Na^+ ^measurement in the 120 min. isoeugenol were outside the I-STAT instrument detection limit (>180 mmol/L Na^+^), and for these samples 180 mmol/L are used. No significant differences were found between the control group and the treated groups for glucose, although there is a trend suggesting increased glucose level in the 120 min. isoeugenol group (Figure 2A). The cortisol increased in both groups after 30 min., and returned to control levels after two hours. Analyzed with Dunn's post-hoc test, this difference was not found to be significant, due to the low number of individual measurements (Figure 2B). The osmolality was significantly increased in the 30 min. isoeugenol- (*P *< 0.05), the 120 min. metacaine- (*P *< 0.05) and in the 120 min. isoeugenol (*P *< 0.001) groups compared to the control group (Figure 2C). Also for Na^+ ^there was a significant increased concentration in the blood after 120 min. in the metacaine- (*P *< 0.01) and isoeugenol (*P *< 0.001) groups (Figure 2D). Both pCO_2 _and HCO_3 _levels were significantly increased in the metacaine and the isoeugenol groups after 120 min. (Figures 2E and 2F). The significance levels were *P *< 0.001 and *P *< 0.01 in the 120 min. metacaine group and P < 0.05 and *P *< 0.001 in the isoeugenol groups. The pH appeared to be increased in the 120 min. isoeugenol group compared to the control group, but the difference was not found to be significant (Figure 2G). Blood hematocrit levels showed no differences between the groups (data not shown).

### RNA quality

**Figure 3:**
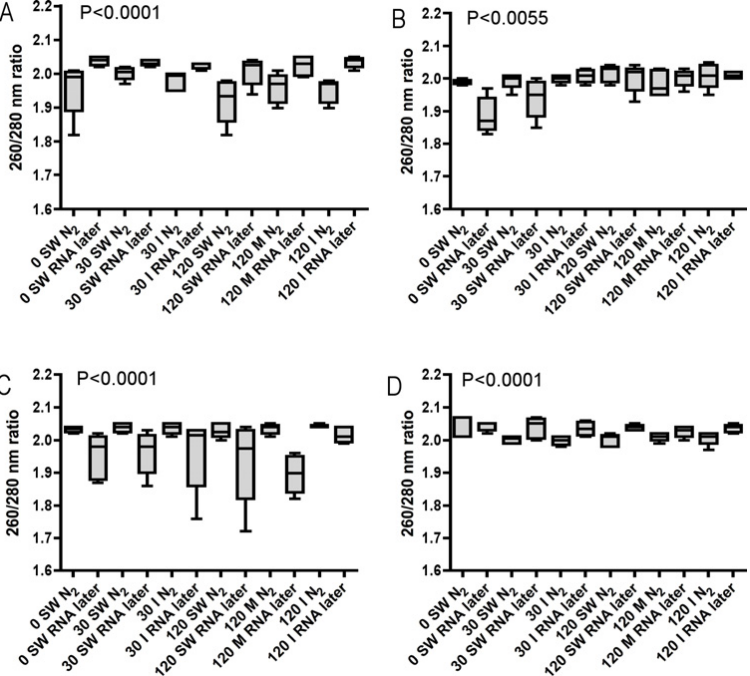
NanoDrop RNA quality assessment (Abs 260/280 nm ratio) of A) liver, B) gills, C) head kidney and D) brain tissues of Atlantic salmon either flash frozen in liquefied nitrogen (N_2_) or preserved in RNA*later *immediately after sampling in the six treatment groups. n = 6 in all groups except 120 min. metacaine and 120 min. isoeugenol groups, n = 5. Group identity: 0 min. seawater control (0 SW), 30 min. seawater (30 SW), 30 min. isoeugenol (30 I), 120 min. seawater (120 SW), 120 min. metacaine (120 M) and 120 min. isoeugenol (120 I). Overall Kruskal-Wallis P-values are shown in the figures.

The different anesthetic treatments described in this work don't have any distinct effects on RNA quality assessed by spectrophotometri with the NanoDrop (Figure 3). Rather the overall Kruskal-Wallis 260/280 nm ratio P-values for all four examined tissues suggest that how fish specimens are preserved significantly affect the quality of RNA extracted from fish tissues. In general, the 260/280 nm ratio assessments suggest that the best way to stabilize and protect RNA in fresh fish liver (Figure 3A) specimens is to use RNA*later*, while freezing samples immediately in N_2 _is the best way to stabilize and protect RNA in gill (Figure 3B) and head kidney (Figure 3C) specimens. For brain tissue (Figure 3D), no conclusive conclusions can be drawn. Analyzing the individual spectrophotometer data (n = 272), a significant correlation was found between the 260/280 and 260/230 nm ratios (Spearman rank correlation, *P *< 0.0001) (data not shown). Low 260/280 nm ratios correlates with low 260/230 nm ratios.

**Figure 4:**
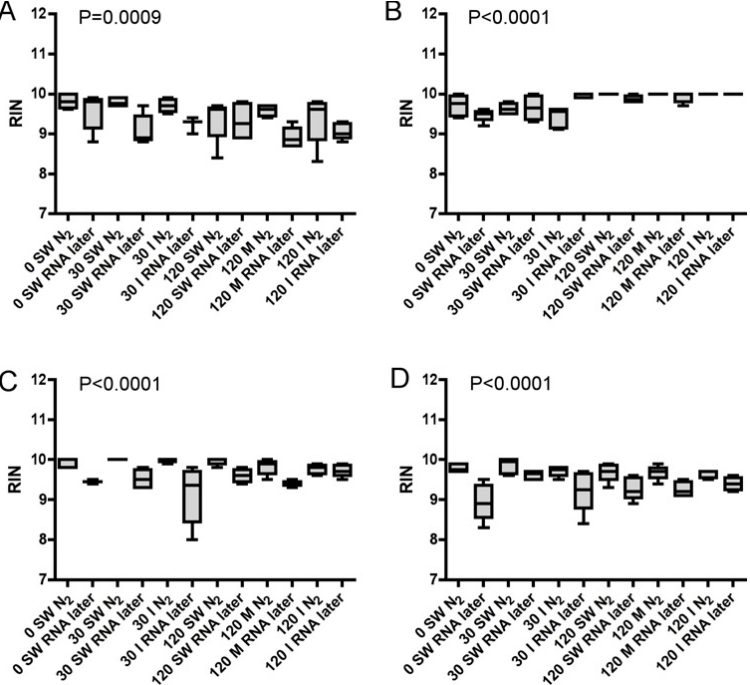
Bioanalyzer RNA quality assessment (RNA integrity number, RIN) of A) liver, B) gills, C) head kidney and D) brain tissues of Atlantic salmon either flash frozen in liquefied nitrogen (N_2_) or preserved in RNA*later *immediately after sampling in six treatment groups. n = 6 in all groups except 120 min. metacaine and 120 min. isoeugenol groups, n = 5. Group identity: 0 min. seawater control (0 SW), 30 min. seawater (30 SW), 30 min. isoeugenol (30 I), 120 min. seawater (120 SW), 120 min. metacaine (120 M) and 120 min. isoeugenol (120 I). Overall Kruskal-Wallis P-values are shown in the figures.

**Figure 5:**
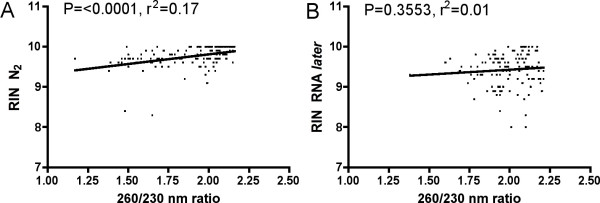
Correlation between individual Abs 260/230 nm ratios and RIN in samples preserved in A) liquefied nitrogen (N_2_) and B) RNA*later*. P-values and correlation coefficients from Spearman rank correlation analysis are given in the graphs. n = 136.

RNA quality analyzed with the RIN tool is shown in Figure 4. The different anesthetic techniques used in this experiment do not seem to have any biased impact on the RNA integrity assessed with the Bioanalyzer. The RIN data show that it is better to flash freeze liver (Figure 4A), head kidney (Figure 4C) and brain tissue (Figure 4D) specimens in N_2 _than to stabilize RNA in RNA*later*. For gill tissue (Figure 4B) there are only minor differences between the two preservation techniques. Average 28S/18S ratio was 1.61 ± 0.27 (n = 272). Potential correlations between individual measurements made with the NanoDrop, i.e. 260/280 and 260/230 nm ratios, and the Bioanalyzer, i.e. and 28S/18S and RIN, were examined with Spearman rank correlation. No significant correlations were found between 260/280 nm ratios and RIN, neither in samples preserved in N_2 _or RNA*later*. A significant correlation between the 260/230 nm ratios and RIN were found in specimens preserved in N_2 _(Figure 5A), but not in specimens preserved in RNA*later *(Figure 5B).

### Gene transcription

**Figure 6:**
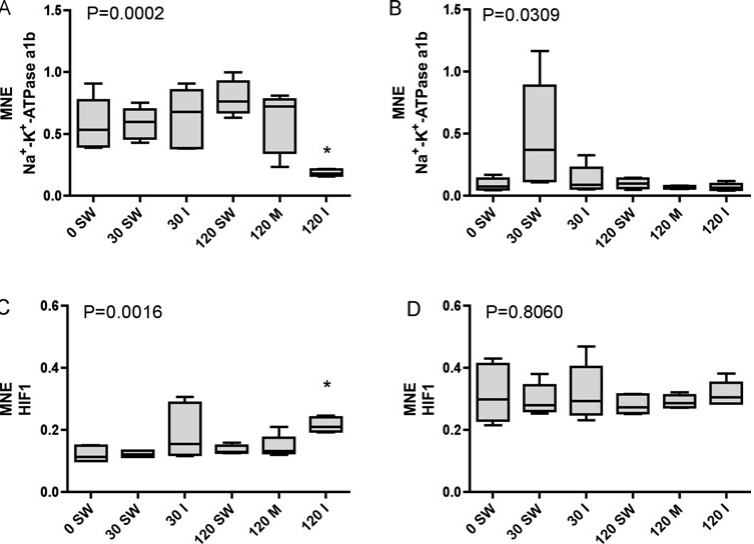
Transcriptional levels (mean normalized expression MNE) of *Na*^+^-*K*^+^-*ATPase α1b *in A) gill and B) brain and of *Hypoxia Inducible Factor 1 *(*HIF1*) in C) gill and D) brain tissues of Atlantic salmon differentially treated during sampling. n = 6 in all groups except n = 5 for *HIF1 *120 min. metacaine, *HIF1 *120 min. isoeugenol and *Na*^+^-*K*^+^-*ATPase α1b *120 min. groups. For the *HIF1 *120 min. isoeugenol group, n = 4. Group identity: 0 min. seawater control (0 SW), 30 min. seawater (30 SW), 30 min. isoeugenol (30 I), 120 min. seawater (120 SW), 120 min. metacaine (120 M) and 120 min. isoeugenol (120 I). An * denotes significant differences (P < 0.05) between the 0 min. control group and the other groups (Kruskal Wallis ANOVA).

Transcriptional levels of two genes known to respond fast after external stimuli's in gill and brain tissues are shown in Figure 6. Mean normalized expression of *Na*^+^-*K*^+^-*ATPase α1b *in gill and brain tissues are shown in Figure 6A and Figure 6B, respectively. *Na*^+^-*K*^+^-*ATPase α1b *was significantly downregulated in the 120 min. isoeugenol group as compared to the control group (*P *< 0.01). In brain tissue no significant differences were found between the groups, although the co-variation (CV) was high in the 30 min. pure seawater group (CV = 88%). *HIF1 *was significantly upregulated in the 120 min. isoeugenol group in gill tissue (Figure 6C), but not in brain tissue (Figure 6D) (*P *< 0.01). The *geNorm *applet was used to calculate mean normalized expression for *Na*^+^-*K*^+^-*ATPase α1b *and *HIF1 *in gill and brain tissues. A normalization factor was found based on the expression of β-*actin*, *EF1A*_*B *_and *ARP*. In gill tissue, β-*actin *was the most stabile gene, with an M value of 0.36, as compared to 0.38 for *EF1A*_*B *_and 0.44 for *ARP*. In brain tissue the corresponding M values were 0.40, 0.37 and 0.40, respectively, with *EF1A*_*B *_being the most stabile gene.

## Discussion

Physiological responses of fish to environmental stressors have traditionally been divided into primary and secondary. Primary responses, i.e. increase in corticostereoid and catecholamine hormones, are fast in teleostean species, with raised plasma cortisol levels observed only minutes after external stimuli. Circulating levels of cortisol have therefore commonly been used as an indicator of the degree of stress experienced by fish [8,9]. Secondary stress include metabolic (i.e. glucose), hematological (i.e. hematocrit) and hydromineral (i.e. Na^+^, plasma osmolality) responses [10]. Both primary and secondary stress parameters measured in this study suggest that fish handling and treatment resulted in a stress response. Mean plasma cortisol level increased from 14.5 ng in the control group to 23.6 ng in the 30 min. pure seawater group (*P *= 0.0087, Mann-Whitney U-test). Cortisol level was also increased in the 30 min. isoeugenol group (22.4 ng), before dropping to levels near the control in all three groups collected after 2 hours. The cortisol levels were somewhat higher than previous measured levels in pre-stressed salmonid fishes, whereas they suggest that the fish were moderately stressed after 30 min. [10]. The increased cortisol levels in both groups after 30 min. contradicts earlier results that showed that 16 mg/L eugenol prevented a surge in cortisol levels compared to stressed fathead minnows *Pimephales promelas *[11]. Small [12] reported that fish anesthetized with eugenol do not have a significant increase in blood cortisol concentrations. Iversen et al. [7] showed that 20 mg/L isoeugenol blocked a cortisol surge in plasma of Atlantic salmon. In rainbow trout, *O. mykiss*, 20 mg/L isoeugenol did not completely eliminate the stress response after crowding and netting [13]. The blood gas parameters suggest that fish handling, especially treatment with isoeugenol for 2 hours, imposed a metabolic alkalosis in the examined animals. A metabolic alkalosis is an acid-base disorder, which causes the plasma bicarbonate (HCO_3_) to rise to a level higher than expected, characterized by a primary increase in HCO_3 _concentration and a compensatory increase in Pa CO_2 _[10]. Increased blood Na^+^, glucose and pH, like observed in the 120 min. isoeugenol group, can also be associated with a metabolic alkalosis. Similar blood metabolic alkalosis has been observed in numerous of salmonids during adaptation to environmental hypercapnia [14,15].

Large-scale sampling of fish tissues under extensive experimental trials can be daunting. In addition to blood and plasma sampling, often a long number of tissue specimens are collected from each individual. As speed of sampling is of pivotal importance in order to obtain high quality RNA from tissue specimens, it is crucial to handle the fish in an appropriate way. Gene transcription patterns can become altered by the handling stress imposed on the fish before sedation and tissue sampling. A stress response can involve a dramatic change in gene expression, commonly resulting in a mass shut-off of transcription [16]. In case, this can trigger alterations in mRNA to total RNA ratios and hence the properties of RNA. Therefore, both how the fish is handled before tissue collection, and how the tissue specimens are preserved may affect transcription patterns in experimental animals. In order to measure transcript levels, high quality RNA has to be purified from tissue specimens. RNA molecules respond rapidly to external stimuli, and are easily degraded. One of the most critical steps in gene transcription studies is therefore to stabilize RNA and preventing RNase enzymes degrading the RNA during tissue sampling and preservation. Until recently, the most common way to stabilize RNA has been to snap freeze tissue specimens; cutting off pieces of tissue and quickly drop them in liquid nitrogen in a cryo tube or wrapped in aluminum. Chemical stabilization of RNA has become more common in recent years. Products like RNA*later *(Ambion) stabilize RNA and can be used to preserve tissue samples, especially when sampling under field conditions. The results from the current examination suggest that overall RNA quality is not affected by physiological stress or by anesthesia treatment of the fish during sampling. As expected, the physiological state of the animals does not have any effects on RNA quality, as long as the tissue cells are in their exponential growth condition at harvesting. The way of stabilizing RNA, however, seems to have an effect on the obtained RNA quality. Assessed by both the NanoDrop and the Bioanalyzer, snap freezing of tissue specimens seem superior to using RNA*later *for most of the studied organs. Only small differences were seen in 260/280 nm ratios between the groups and treatments. Both RNA preservation techniques gave 260/280 nm ratios between 1.8 and 2.0. Especially for head kidney tissue, RNA*later *preservation seems to yield more variable 260/280 nm ratios as compared to specimens flash frozen in liquefied nitrogen. One natural explanation for this finding is that head kidney specimens are normally sampled last when collecting tissue specimens from fish. The data suggest that RNA*later *penetrates head kidney tissue slower than for example liver tissue, allowing RNases to start chewing RNA. In mammals, contradicting findings have been reported, suggesting that kidney specimens are best preserved with RNA*later *[17]. Obtained with the Bioanalyzer, RIN between 9 and 10 were achieved from all six treatments groups and both preservation techniques, signaling high quality RNA. Recently, Fleige and Pfaffl [18] recommended a RIN higher than eight as perfect total RNA for downstream applications like qRT-PCR. In general, the results show that flash freezing technique give slightly better RNA quality than RNA*later *preservation, with higher RIN and lower CV's. Analyzing the individual data, the most surprising finding was that there was a significant correlation between the 260/230 nm ratios and RIN in specimens preserved in N_2_, but not in specimens preserved in RNA*later*. At present, the reason for this finding is unknown.

mRNA half-lives range form 15 min. to more than 10 hours in mammals [19]. In order to check if prolonged sedation for 120 min. affects mRNA transcription, qRT-PCR assays for two genes known to respond fast to external stimuli's were developed (*Na*^+^-*K*^+^-*ATPase α1b *[20,21] and *HIF1 *[22]. In gills, but not in brain tissue, isoeugenol sedation for 120 min. resulted in a significantly downregulation of the *Na*^+^-*K*^+^-*ATPase α1b *gene and a significantly upregulation of the *HIF1 *gene, clearly suggesting that choice of sedation during sampling might affect transcription patterns in fish. Na^+^-K^+^-ATPase plays a central role in ionoregulation in teleosts, as it maintain Na^+ ^and K^+ ^gradients across the basolateral membrane in fish gills. Four α-subunit isoforms of Na^+^-K^+^-ATPase have been described in rainbow trout gill [20]. The *Na*^+^-*K*^+^-*ATPase α1b *isoform studied here has been shown to increase following seawater exposure. Altered *Na*^+^-*K*^+^-*ATPase α1b *expression in the 120 min. isoeugenol group indicates that these individuals have experienced perturbation in ionoregulation. In mammals, the transcription factor HIF1 is a heterodimer consisting of two subunits, HIF1α and HIF1β [23]. The inducible HIF1 form studied here, probably a HIF1α homolog, mediates the expression of a series of genes involved in both cellular and systemic responses to hypoxia, leading to enhanced anaerobic metabolism [22]. Altered *HIF1 *transcription signals that fish in the 120 min. isoeugenol group have experienced hypoxic stress, even though the water in the crowding tank was oxygenated throughout the experimental period.

## Conclusion

Physiological stress and choice of sedation/anesthetics during fish sampling do not seem to affect the quality of RNA extracted from liver, gill, head kidney and brain tissue specimens of Atlantic salmon preserved in N_2 _or RNA*later*. 120 min. sedation of juvenile Atlantic salmon in 5 mg/L isoeugenol affected the transcriptional levels of two genes involved in ionoregulation and respiration, suggesting that prolonged use of sedation and anesthetics should be carefully applied when sampling fish specimens for gene transcription analysis. In general, the results also suggest that for tissue preservation it is better to flash-freeze fish specimens in liquefied N_2 _than to use RNA*later*.

## Methods

### Fish handling

About 130 seawater-adapted Atlantic salmon with an average weight of 350 grams were obtained from the Matre Aquaculture Research Station in April 2006, and kept in a 1500 L tank at the Institute of Marine Research, Bergen, Norway, until sampling by the end of June. Figure 1 shows an overview of the experimental design. A total of 36 individuals were used in the experiment. All fish were starved for 24 hours before the start of the experiment. Six fish were sampled directly from the outdoor 1500 L storage tank at time zero. These individuals were not treated by any sedative or anesthetics, and were killed by a blow to the head. Then 24 fish were moved into two smaller tanks (12 individuals in each) and another six fish into a third tank, each containing about 80 L of seawater. Six individuals were sampled from each of the two tanks containing 12 fish after 30 min. The seawater in one of the tanks was added 10 mg/L Aqui-S (5 mg/L isoeugenol) (Aqui-S New Zealand Ltd, Lower Hutt, New Zealand) sedative from time zero, whereas the other tanks only contained pure seawater. For sedation of salmonids, it is recommended using doses between 10–25 mg/L Aqui-S. Used as an anesthetic, recommended doses are between 50–100 mg/l Aqui-S. After two hours, six fish were sampled from each of the three tanks. Fish in tank one were treated with isoeugenol for two hours, fish in tank two were kept in pure seawater for about 1 hour and 45 min., given a 15 min. anesthetic treatment with 100 mg/L metacaine (Norsk Medisinaldepot, Oslo, Norway) and then killed by a blow to the head. Fish in tank three were killed directly by a blow to the head without any anesthetic treatment. The fish were weighing on average 455 ± 80 g when sampled. All three tanks were oxygenated throughout the two-hour experimental period. The oxygen levels never dropped below 100% in neither of the tanks, measured with a WTW (Oxi 315i, WTW, Weilheim, Germany). Water temperature was 9.3°C in the outdoor tank at time zero, and the temperature rose to a maximum of 10.5°C in the 80 L tanks after two hours of exposure.

### Tissue sampling

Five tissues, liver, gills, head kidney, brain and lens, were sampled June 30^th ^2006 from a total of 36 Atlantic salmon with an average weight of 455 ± 80 g. The tissue samples were dissected out as fast as possible, and sliced into two parts (liver, head kidney and brain). One part was immediately frozen in liquefied nitrogen and the other part preserved in RNA*later *(Ambion Inc., Austin TX, USA). 50–80 mg of tissue was preserved in 1 ml RNA*later (>1/10)*. The RNA*later *samples were kept on ice after sampling, stored overnight at 4°C, and kept frozen at -20°C until RNA extraction. The soft part of the gills were sliced off the gill arch and split into two parts. For the lenses, one was frozen in liquefied nitrogen and the other stored in RNA*later*. Data from the lens study will be described in a separate work.

### Blood parameters

Blood samples were taken from the fish just after anesthetization from the caudal vein. One ml of whole blood was sampled from each fish in 1 ml syringes, containing about 30 μl heparin (5000 IE/a.e./ml, LEO Pharma A/S, Oslo, Norway), and kept on ice until plasma separation. Whole blood ion (Na^+^), glucose, pH and gas parameters (pCO_2_, HCO_3_) were analyzed by a clinical I-STAT analyzer using CG8^+ ^cartridges (I-STAT Corporation, Windsor, USA). Hematocrit was measured with a Haemofuge A centrifuge (Heraeus-Christ GmbH, Osterode am Harz, Germany). Plasma was separated from the blood by centrifugation (2000 g × 10 min.). Plasma cortisol levels were determined using a radioimmunoassay (RIA) (125I cortisol kit, Bio-Rad Laboratories, Richmond, CA, USA). Osmolality levels in the plasma were determined using a Fiske One-Ten Osmometer (Fiske Associates, MA, USA).

### RNA extraction

Tissues were thoroughly homogenized before RNA extraction with zirconium beads (4 mm) in a Retsch MM 301 homogenizer (Retsch GmbH, Haan, Germany). Total RNA was extracted using Trizol reagent (Invitrogen, Life Technologies, Carlsbad, CA, USA), according to the manufacturer's instructions and stored in 100 μl RNase-free MilliQ H_2_O. Genomic DNA was eliminated from the samples by DNase treatment according to the manufacturer's description (DNA-*free*, cat. no. 1906, Ambion, Austin, TX, USA). The RNA was then stored at -80°C before further processing.

### RNA quality and integrity

The quality of the RNA was assessed with the NanoDrop^® ^ND-1000 UV-Vis Spectrophotometer (NanoDrop Technologies, Wilmington, DE, USA) and the Agilent 2100 Bioanalyzer (Agilent Technologies, Palo Alto, CA, USA) according to the manufacturer's instructions. 260/280 and 260/230 nm absorbance ratios of 1.8 – 2.0 indicate a pure RNA sample. The RNA 6000 Nano LabChip^® ^kit (Agilent Technologies, Palo Alto, CA, USA) was used to evaluate the integrity of the RNA. A 28S/18S rRNA ratio of 1.8–2.0 signals undegraded RNA. The RNA integrity number (RIN) is a software tool developed by Agilent designed to help scientists estimate the integrity of total RNA samples. The software automatically assigns an integrity number to a eukaryote total RNA sample run on a Bioanalyzer RNA chip. A RIN of 10 signals a pure RNA sample [2].

### Quantitative real-time RT-PCR

For gene transcription analysis RNA extracted from tissue samples preserved in N_2 _was used. The PCR primer sequences used for quantification of the genes encoding β-*actin*, *elongation factor 1 alpha *(*EF1A*_*B*_), *acidic ribosomal protein *(*ARP*), *Na*^+^-*K*^+^-*ATPase alpha subunit 1b *(*Na*^+^-*K*^+^-*ATPase α1b*) and *hypoxia inducible factor 1 *(*HIF1*) are shown in Table 1. PCR primers for β-*actin *were based on Atlantic salmon [Genbank: BG933897] and designed to span exon-exon borders of this gene, as deduced from corresponding genes in human and zebrafish. The *EF1A*_*B *_assay was based on the EST [Genbank: AF321836], also spanning exon-exon borders. These two reference genes have also been used as references in real-time RT-PCR analyses of Atlantic salmon in other recent studies [24,25]. The mRNA sequences encoding *ARP *and *HIF1 *were obtained from GenBank accession numbers [Genbank: AY255630] and [Genbank: AF304864], respectively (exon-exon borders were not considered). The *ARP *primer pair was obtained from an *Oncorhynchus tshawytscha *sequence, and the *HIF1 *primer pair from an *Oncorhynchus mykiss *sequence. *ARP *has previously been shown to a promising qRT-PCR reference gene in Atlantic salmon [26]. PCR primers for *Na*^+^-*K*^+^-*ATPase α1b *were obtained from accession number [Genbank: AY319390], an *O. mykiss *primer pair for this gene described by Richards et al. [20] that amplified a PCR product also in Atlantic salmon (verified with a One-Step RT-PCR kit from Qiagen (Qiagen, Chatsworth, CA, USA) and run on a 2% agarose gel). The primer pairs amplify PCR products between 59–106 basepairs (bp) long, which is within the range of 50–150 bp as suggested by Applied Biosystems for their TaqMan assays. However, in this study SYBR Green real-time PCR chemistry was applied, without the use of sequence-specific probes. qPCR assays were designed using Primer Express 2.0 software (Applied Biosystems, Foster City, CA, USA) (except for *Na*^+^-*K*^+^-*ATPase α1b*) to select appropriate primer sequences from known salmonid genes. RNA samples were subjected to DNase treatment to avoid genomic DNA contamination, since three of the assays didn't span exon-exon borders. Amplified PCR products of all actual cDNAs were sequenced to ensure that the correct mRNA sequences were quantified. The fragments were sequenced with BigDye version 3.1 fluorescent chemistry (Applied Biosystems) and run on an ABI PRISM^® ^377 DNA apparatus at the University of Bergen Sequencing Facility.

**Table 1:** PCR primers and amplicons

**Gene**	**Forward primer**	**Reverse primer**	**Amplicon (bp)**
Beta-actin	CCAAAGCCAACAGGGAGAA	AGGGACAACACTGCCTGGAT	92
EF1AB	TGCCCCTCCAGGATGTCTAC	CACGGCCCACAGGTACTG	59
ARP	TCATCCAATTGCTGGATGACTATC	CTTCCCACGCAAGGACAGA	101
Na+/K+-ATPase alpha 1b	CTGCTACATCTCAACCAACAACAT	CACCATCACAGTGTTCATTGGAT	81
HIF1	CCACCTCATGAAGACCCATCA	TCTCCACCCACACAAAGCCT	101

Number of base pairs (bp) are given for each amplicon.

A two-step real-time RT-PCR protocol was developed to measure the mRNA levels of the five genes in gill and brain tissues of Atlantic salmon. The RT reactions were run in duplicate on 96-well reaction plates with the GeneAmp PCR 9700 machine (Applied Biosystems, Foster City, CA, USA) using TaqMan Reverse Transcription Reagent containing Multiscribe Reverse Transcriptase (50 U/μl) (N808-0234, Applied Biosystems, Foster City, CA, USA). Twofold serial dilutions of total RNA were made for efficiency calculations. Six serial dilutions (1000 – 31 ng) in triplicates were analyzed by qRT-PCR in separate sample wells and the resulting Cts recorded. Total RNA input was 500 ng in each reaction for all genes. No template control (ntc) and RT-control (a duplicate RNA sample analysis where only the RT enzyme is left out) reactions were run for quality assessment. RT-controls were not performed for every individual sample, but were run for each assay or gene, with the same sample as used to make the dilution curves on the 96 well plates. Reverse transcription was performed at 48°C for 60 min by using oligo dT primers (2.5 μM) for all genes in 30 μl total volume. The final concentration of the other chemicals in each RT reaction was: MgCl2 (5.5 mM), dNTP (500 mM of each), 10 × TaqMan RT buffer (1 ×), RNase inhibitor (0.4 U/μl) and Multiscribe Reverse Transcriptase (1.67 U/μl).

2.5 μl cDNA from each RT reaction for all genes was transferred to a new 96-well reaction plate, and the real-time PCR run in 25 μl reactions on the ABI Prism 7000 Sequence Detection System from AB. Real-time PCR was performed by using SYBR Green Master Mix (QuantiTect SYBR Green PCR kit, cat. no. 204143, Qiagen, Chatsworth, CA, USA), which contains HotStar Taq DNA polymerase and gene specific primers (300 nM). PCR was achieved with a 15 min activation and denaturation step at 95°C, followed by 40 cycles of 15 s at 95°C and 60 s at 60°C. Baseline and threshold for Ct calculation were set automatically with the ABI Prism 7000 SDS software version 1.1, or set manually whenever necessary. The *geNorm *VBA applet for Microsoft Excel was used to determine a normalization factor from the three examined reference genes used to calculate mean normalized expression (MNE) for *Na*^+^-*K*^+^-*ATPase a1b *and *HIF1 *[27]. The Ct values were transformed to quantities using standard curves, according to the *geNorm *manual. *geNorm *determines the individual stability of a gene within a pool of genes, and calculates the stability according to the similarity of their expression profile by pair-wise comparison, using the geometric mean as a normalizing factor. The gene with the highest M, i.e. the least stabile gene, is then excluded in a stepwise fashion until the most stabile genes are determined. Here a normalizing factor based on all three examined reference genes was used to calculate the MNE.

### Statistics

The GraphPad Prism 4.0 software (GraphPad Software, Inc.) was used for the statistical analyses in this work. Nonparametric Kruskal-Wallis test was used to compare differences among the six groups of salmon examined. Dunn's multiple comparison post-test was used to compare the different groups. Spearman rank correlation and linear regression were used to determine correlation coefficients in comparing individual spectrophotometer and RNA integrity data. An alpha level of 0.05 was considered significant.

## Authors' contributions

PAO initiated the research and was responsible for all parts of the project. He also wrote the paper. KKL participated in planning, sampling, experimental analysis and critically evaluated the manuscript. EMH participated in planning, sampling and evaluation of the manuscript.
